# Understanding the Cytomegalovirus Cyclin-Dependent Kinase Ortholog pUL97 as a Multifaceted Regulator and an Antiviral Drug Target

**DOI:** 10.3390/cells13161338

**Published:** 2024-08-13

**Authors:** Manfred Marschall, Martin Schütz, Markus Wild, Eileen Socher, Christina Wangen, Kishore Dhotre, William D. Rawlinson, Heinrich Sticht

**Affiliations:** 1 Institute for Clinical and Molecular Virology, Friedrich-Alexander-Universität Erlangen-Nürnberg (FAU), 91054 Erlangen, Germany; martin.schuetz1@web.de (M.S.); markus-wild@mail.de (M.W.); christina.wangen@uk-erlangen.de (C.W.); kishore.dhotre@uk-erlangen.de (K.D.); 2 Institute of Anatomy, Functional and Clinical Anatomy, Friedrich-Alexander-Universität Erlangen-Nürnberg, 91054 Erlangen, Germany; eileen.socher@fau.de; 3 Serology and Virology Division, NSW Health Pathology Microbiology, Prince of Wales Hospital, and Schools of Biomedical Sciences, Women’s and Children’s Health, Medicine and Biotechnology and Biomolecular Sciences, University of New South Wales, High Street, Sydney 2050, Australia; w.rawlinson@unsw.edu.au; 4 Division of Bioinformatics, Institute of Biochemistry, FAU, 91054 Erlangen, Germany; heinrich.sticht@fau.de

**Keywords:** herpesviral protein kinases, human cytomegalovirus, cytomegaloviral vCDK/pUL97, regulation of viral replication, impact of vCDK/pUL97, cyclin H and CDK7, antiviral approaches with kinase inhibitors, novel targeting strategies

## Abstract

Herpesviral protein kinases, such as the therapy-relevant pUL97 of human cytomegalovirus (HCMV), are important for viral replication efficiency as well as pathogenesis, and represent key antiviral drug targets. HCMV pUL97 is a viral cyclin-dependent kinase (CDK) ortholog, as it shares functional and structural properties with human CDKs. Recently, the formation of vCDK/pUL97–cyclin complexes and the phosphorylation of a variety of viral and cellular substrate proteins has been demonstrated. Genetic mapping and structural modeling approaches helped to define two pUL97 interfaces, IF1 and IF2, responsible for cyclin binding. In particular, the regulatory importance of interactions between vCDK/pUL97 and host cyclins as well as CDKs has been highlighted, both as determinants of virus replication and as a novel drug-targeting option. This aspect was substantiated by the finding that virus replication was impaired upon cyclin type H knock-down, and that such host-directed interference also affected viruses resistant to existing therapies. Beyond the formation of binary interactive complexes, a ternary pUL97–cyclin H–CDK7 complex has also been described, and in light of this, an experimental trans-stimulation of CDK7 activity by pUL97 appeared crucial for virus–host coregulation. In accordance with this understanding, several novel antiviral targeting options have emerged. These include kinase inhibitors directed to pUL97, to host CDKs, and to the pUL97–cyclin H interactive complexes. Importantly, a statistically significant drug synergy has recently been reported for antiviral treatment schemes using combinations of pharmacologically relevant CDK7 and vCDK/pUL97 inhibitors, including maribavir. Combined, such findings provide increased options for anti-HCMV control. This review focuses on regulatory interactions of vCDK/pUL97 with the host cyclin–CDK apparatus, and it addresses the functional relevance of these key effector complexes for viral replication and pathogenesis. On this basis, novel strategies of antiviral drug targeting are defined.

## 1. Aspects of Human Herpesvirus Pathogenesis and Antiviral Therapy

### 1.1. General Features of Human Pathogenic Herpesvirus Infections

The virus family *Herpesviridae* includes important human and animal pathogens. They present with diverse symptoms of infection, signs, and clinical outcomes, including lethal courses, tumors, and immunological disorders [1]. The type species of the three herpesviral subfamilies α-, β-, and γ-*Herpesvirinae* are represented by herpes simplex virus 1 (HSV-1), human cytomegalovirus (HCMV), and Epstein-Barr virus (EBV), respectively. Herpesvirus infections persist for the entire lifetime of the individual in a latent state, and no current antiviral is able to eliminate herpesviral latency in the human host. Clinical disease occurs on primary infection, or more often with virus reactivation during host immune suppression. Infection may cause virus-specific malignancies over long-term infection, and is best demonstrated for the γ-herpesviruses (EBV and Kaposi’s sarcoma-associated herpesvirus, KSHV), although tumor-relevant events of viral host interaction are also hypothesized for α- or β-herpesvirus infections.

Infections with HSV-1/-2 (α-herpesviruses) result in recurrent orolabial or genital pa-thology and visceral disease in immunocompromised patients. Reactivation of the α-herpesvirus varicella zoster virus (VZV) results in zoster (shingles) in immunocompetent and immunocompromised individuals, and organ disease in immunocompromised patients. Reactivation of the β-herpesvirus HCMV can cause severe organ disease in immunosuppressed transplant recipients and other patients with immune compromise from disease, such as cancer or connective tissue disease, and immune-suppressing therapies. Primary infection, reinfection, and most commonly, reactivation of latent infection, all contribute to significant morbidity and mortality in recipients of stem cell and solid organ transplants [2,3,4,5]. Of specific note, congenital HCMV infection (cCMV) is the most frequent infection-based risk during pregnancy. Thus, cCMV represents a serious medical problem, frequently leading to developmental defects of the unborn and neonates, particularly upon generalized virus infection [6]. The γ-herpesvirus EBV is associated with several B cell lymphomas and epithelial cell carcinomas (nasopharyngeal carcinoma, NPC), lymphoepithelioma-like carcinoma or post-transplant lymphoproliferative disease (PTLD), as well as EBV-associated gastric cancers (EBVaGC). Recently the evidence for EBV association with multiple sclerosis (MS) provides the opportunity for trials of novel MS therapies, including EBV vaccination and antiherpesviral drugs, to reduce the effect of this cofactor [7,8,9].

Although numerous clinical trials of therapeutic vaccines have been performed to prevent reactivation of HSV, HCMV, and EBV, none of these has yet proven of sufficient efficacy to be licensed. The β-herpesvirus HCMV provides particular epidemiological and pathogenetic issues to address, as a major human pathogen with worldwide distribution, and given the large amount of HCMV-directed host immune response in humans and in animal models. Most HCMV infections are asymptomatic in the immunocompetent. However, severe or even life-threatening sequelae may occur in immunosuppressed individuals or the immunonaïve fetus and neonate. Current therapies are inadequate in these groups, meaning new therapies need to be developed using licensed and novel antivirals. As a striking feature, HCMV-induced pathogenesis in the immunocompromised host is widely determined by the magnitude of viral replication and virulence in the host [10]. This means that specific cytomegaloviral pathogenic patterns in humans are mostly determined by active virus multiplication in infected tissues, eventually leading to high viral load and viremia. Development and trials of novel drugs against newly recognized targets of HCMV infection is of increasing importance, due to the absence of any approved HCMV vaccine and the increasing selection of HCMVs with resistance to existing antivirals [11,12].

### 1.2. Current Antiherpesviral Therapeutic Options and the First Approved Antiviral Kinase Inhibitor

There is an ongoing need for control of HCMV, particularly in stem cell and solid organ transplant recipient patient populations. This is because the rate of seropositivity in the human population remains high (in high-income countries, 40% in midlife, increasing to 80 to 90% in the elderly), the higher rates of seropositivity in low-middle-income countries (LMIC, approaching 100%) and the lifelong persistence of HCMV in the seropositive human host. The reactivation, reinfection, as well as less frequent primary infection, of HCMV in transplant recipients remains the single most frequent, and in many ways the most important, complication of transplantation [13]. Antiviral prophylaxis following transplantation has enormously reduced the severity and complications in this population resulting from HCMV infection and reactivation. However, there is an increasing number of immunocompromised patients surviving longer terms globally. This is due to increased numbers of transplants, as well as the more frequent use of effective immunosuppressive agents in a greater range of patients. Ganciclovir (GCV) and the oral prodrug valganciclovir (VGCV) were enormous advances in control of HCMV in these populations. GCV, available since the mid-1980s, unfortunately has significant adverse effects. In particular, these can result in partially reversible bone marrow suppression, which is important in transplant recipients, where neutropenia and other infections are of high clinical significance, and in known effects on the gonads in animal studies. These adverse effects make the use of GCV and VGCV in the human neonatal population and among pregnant women of significant concern. Alternative treatments for HCMV such as foscarnet, cidofovir, and brincidofovir (the latter of which is currently not available) all have serious adverse effects and are typically used intravenously. Other non-pharmacologic treatments, including intravenous immunoglobulin (IVIG, either standard or HCMV-specific), have been used, although these are typically not regarded as well-proven or efficacious in HCMV treatment. IVIG has been used as adjunctive therapy in HCMV prophylaxis [14] and disease treatment [15]. Most published use has been in heart and lung transplant recipients, particularly D+/R− transplants in 10–40% of cases in the USA (reviewed in [16]). The presumed mechanisms of IVIG action include (i) neutralization of free viral particles through enhanced opsonization and phagocytosis, (ii) increased virus lysis by complement- and antibody-mediated responses, and (iii) later enhancement of antibody-dependent cellular cytotoxicity (ADCC) corresponding to the appearance of cytokine-producing CD4+ cells. Systematic reviews of the use of HCMV-specific IVIG in solid organ transplantation (SOT) and meta-analysis of studies did not show improvement in HCMV infection, disease, or other complications (acute rejection, graft loss, opportunistic infection, and mortality [17]. However, meta-analysis of a subset of studies (randomized controlled trials only) suggested prophylaxis using HCMV-IVIG in SOT resulted in improvements of survival and reduced disease without reduction of infections [18]. Thus, in considering the use of antivirals in difficult clinical settings or where resistance is present, IVIG may be considered, generally as an adjunctive therapy [19]. T cell therapies (TCTs), using ex vivo cultured cells reinfused into the HCMV-infected patient, are available in some centres. Most use has been in haematopoietic stem cell transplantation (HSCT), reviewed in [20]. Recent studies of TCT in SOT are limited, and no large-scale studies have been performed. The major issue with TCT is availability and cost, although these factors may be addressed over time. Other issues with TCT include the need for individual-specific T cells, although these may be available off the shelf. The in vitro TCT are highly sensitive to immunosuppressives (CyA and FK506), reduced T cell differentiation in SOT receiving immunosuppression, and a risk of alloreactive T cells coming across with the TCT (reviewed in [20]).

Clinical and genotypic resistance to all of these existing antiviral agents is an ongoing problem, particularly as increased numbers of highly immunosuppressed patients are being treated for longer periods of time. This enhances the risk of the development of antiviral resistance, thus limiting the use of these drugs [21,22,23]. HCMV shows a relatively pronounced capacity to mutate, and the diversity of strains within the infected human host suggests the problem of resistance and refractoriness of HCMV to antivirals will persist [24]. Fortunately, novel antivirals have been developed, and although not the complete answer, they do provide alternative treatments going forward.

Maribavir/Livtencity^®^ (MBV) represents a currently second-tier antiviral that has been shown in a phase III trial to be superior to investigator-assigned therapy of VGCV/GCV, foscarnet, or cidofovir or efficacy in recent trials [2]. The study also achieved secondary endpoints, with minimal adverse events including rare acute kidney injury. The use of MBV has overcome similar problems with other second-line therapies, such as foscarnet [25], and MBV may well become the primary second-line treatment in populations, once available globally. MBV acts on multiple stages of HCMV replication, and thereby shows a dominant inhibitory effect on viral nucleocytoplasmic egress regulation. It is a benzimidazole riboside that strongly inhibits the viral pUL97 protein kinase and the additional effects on other viral proteins (pUL27, and possibly pUL56), meaning that MBV potentially provides a novel way of targeting GCV-resistant strains [6,11,12]. As with other new agents, MBV represents a possible second or even third agent for use in combination with other antivirals [26], as also discussed below. In the real world, treating patients with antiviral agents is complex. Thus, the use of MBV in small numbers of patients has been complicated by relapse, refractoriness, and development of resistance [27]. Earlier studies, and more recent successful phase III studies, have all demonstrated the development of drug-resistant mutations in ORF-UL97, and in some cases ORF-UL27 genes, (both in vitro) and during clinical trials [28,29]. Although resistance is currently infrequent, with more extensive use, particularly in individuals with high levels of immunosuppression and varying levels of blood compliance, it will almost certainly increase [29]. Letermovir/Prevymis^®^ (LMV) is another novel antiviral initially approved in most countries for use as a prophylaxis in HSCT, which has occasionally been used for treatment. However, with licensing of this drug in many more countries, increased use in SOTs has occurred [30,31,32]. Also, against LMV, viral resistance was documented [33]. Interestingly, some authors have used GCV in combination with LMV. This is a mechanistically novel approach, given that GCV acts on the early-late stage of viral genome replication, and LMV acts on the late stage of viral DNA cleavage [34]. Future investigation may clarify the potential clinical benefits of direct-acting anti-HCMV combination treatments.

## 2. Herpesvirus-Encoded Protein Kinases

### 2.1. The Functional Complexity of Herpesviral Protein Kinases

**Figure 1:**
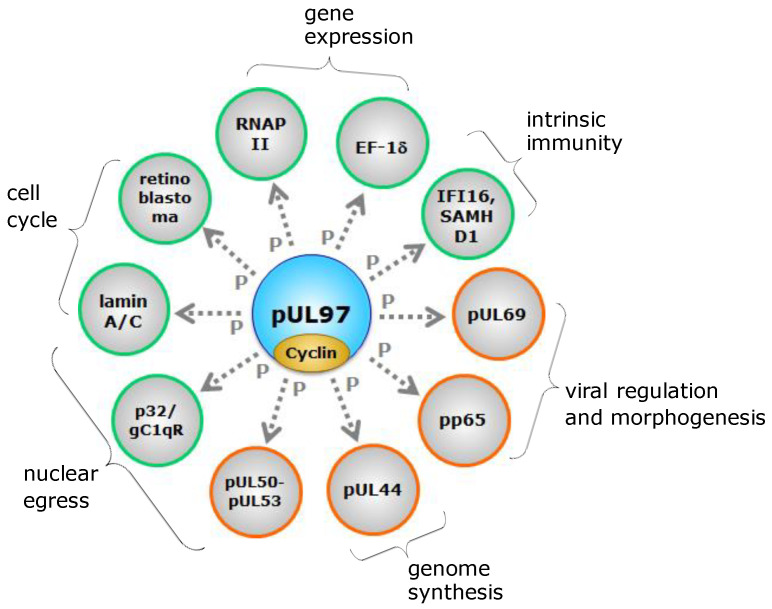
Schematic representation of the HCMV-encoded protein kinase pUL97 and its various substrates. The CDK-like protein kinase pUL97 of human cytomegalovirus interacts with cyclins and phosphorylates (P), as well as a number of viral (encircled in orange) and cellular (encircled in green) substrate proteins. Functional assignment of the individual proteins is indicated by curly brackets. RNAPII, cellular RNA polymerase II; EF-1δ, translational elongation factor 1δ; IFI16, interferon-inducible protein 16; SAMHD1, SAM domain and HD domain-containing protein 1; pUL69, pp65, pUL44, pUL50, pUL53, cytomegaloviral early proteins with specific regulatory functions; p32/gC1qR, acidic 32-kDa multiligand-binding protein as a receptor for globular head domain of complement C1q; lamin A/C, human nuclear lamin protein of types A and C; Rb, human retinoblastoma protein.

Only a limited number of human and animal viral genomes code for their own regulatory protein kinases (PKs), including members of the families *Herpesviridae*, *Poxviridae*, *Retroviridae*, and *Baculoviridae* (reviewed in [35]). Among herpesviruses, two homology groups of viral PKs have been defined, namely the UL group (termed after herpes simplex virus type 1 [HSV-1] and HCMV prototype kinases UL13 and UL97), and the US group (termed after HSV-1 prototype US3). The UL group of herpesviral protein kinases, HvUL, is present in all herpesviruses characterized so far, while the HvUS group is restricted to the α-herpesvirus subfamily. The 11 functionally important sequence motifs typical for serine/threonine kinases are considerably well-conserved in the catalytic domains of herpesviral protein kinases (HvPKs). As such, HvPKs contain a lysine in motif II corresponding to a highly conserved lysine in host protein kinases; any mutation of which results in a complete loss of kinase activity (e.g., mutant K355M of the HCMV pUL97 kinase [36]). The HvPKs characterized so far are apparently not essentially required for minimal levels of viral replication, at least in cell culture, but deletion mutants are severely attenuated for viral replication ([37] and references therein). Typically, HvUL kinases are expressed with early–late kinetics, and in addition, they are incorporated into virions as tegument proteins [38,39]. A number of functions have been demonstrated or postulated for the individual HvUL kinases during viral replication, such as tegument disassembly, modulation of gene expression, stimulation of viral DNA replication, and, importantly, a role in the nuclear capsid egress mediated through a phosphorylation-dependent disruption of the nuclear lamina (Figure 1 [40,41,42]). In particular, the HvUL protein kinase pUL97 is an important determinant of efficient HCMV replication. Deletion of the ORF-UL97 from the viral genome or pharmacological inhibition of the kinase activity results in a rigorous reduction of the virus replication efficiency by a factor of 100–1000 [43,44,45,46]. pUL97 exerts influence on HCMV replication by phosphorylation of both viral and cellular proteins, such as pUL44, pUL69, pp65, nuclear lamins, the multifunctional adapter protein p32, retinoblastoma (Rb) protein, RNA polymerase II, and translational elongation factor EF-1δ (Figure 1; [40] and references therein). Further, pUL97 is expressed in three isoforms, all possessing an autophosphorylation activity that is significantly influenced by the formation of pUL97 homodimers or oligomers via its self-interaction domain [36,47].

Detailed studies have focused on the crosstalk between herpesviral and cellular protein kinases and their functional relationship with each other in the human host. In particular, HCMV replication is restricted to specific host cell types and is dependent on the balance of interactions between viral and cellular proteins. In this regard, various virus- or cell-encoded PKs display crucial functions during HCMV replication and are interlinked in several aspects of their regulatory properties [42,48,49,50]. A very common strategy employed by viruses is the manipulation of the host CDK–cyclin machinery to hijack and control both cellular and viral activities [51]. The cell cycle in uninfected cells is tightly regulated through checkpoints that ensure accurate cell cycle progression. The cell cycle comprises four phases: gap 1 (G1), synthesis (S), gap 2 (G2), and mitosis (M). In addition, differentiated cells usually stay in a quiescent (G0) phase [52]. Normally, external factors, including mitogens, bind to receptors on the cell surface to activate pathways such as the RAS/mitogen-activated protein kinase. This pushes the cells into the G1 phase, in which mRNAs and proteins for DNA synthesis are produced. If the positive proliferative signal continues, cells proceed beyond the G1/S phase border into the S phase, in which the cellular genome is duplicated, irrespective of the presence of the signal. Subsequently, cells transit into the G2 phase to prepare for mitotic cell division and undertake DNA repair, if needed. Once the DNA integrity is verified and the appropriate signals are received, cells progress into the M phase, which leads to the distribution of the replicated chromosomes to two daughter cells (reviewed by [53]).

Unlike smaller DNA viruses, which hijack the cellular DNA replication machinery, herpesviruses encode their own DNA polymerase and accessory factors for replication. Consequently, and this has been intensively studied for HCMV, virus infection induces a cell cycle arrest at the G1/S phase transition, before the expression of the host replication machinery. This may be highly advantageous for HCMV, since it reduces competition for the limited DNA-replication resources. During the lytic phase of infection, HCMV maintains in an active metabolic state within the host cell, termed the early S phase arrest or pseudomitosis [54]. This unique state is characterized by the expression of selected proteins associated with G1, S, and M phases [55]. To achieve this condition, HCMV interferes with the cell cycle at multiple levels, including transcription, post-transcriptional processing, translation, post-translational modification, protein stability, and cellular protein localization [56]. Certain cell cycle-regulating cyclin-dependent kinase (CDK)–cyclin complexes are either activated, such as CDK1–cyclin B1 and CDK2–cyclin E, or downregulated, such as CDK4/6–cyclin D and CDK2–cyclin A. Furthermore, the G1 checkpoint protein retinoblastoma-associated protein (Rb) is targeted for either degradation by the viral protein pp71 or hyperphosphorylation by the viral kinase pUL97 [57,58]. This results in the release of E2F transcription factors, which stimulate the transcription of certain genes important for DNA replication and cell proliferation. Another master regulator of the cell cycle is the E3 ubiquitin ligase anaphase-promoting complex (APC). This complex is crucial for cells to advance through anaphase, exit mitosis successfully, and avoid premature entry into S phase [59]. Research suggests that pUL21a and the viral kinase pUL97 mediate the downregulation of specific APC subunits, leading to its inhibition [56,60,61].

In general, CDKs and cyclins are central regulators of both cell cycle and transcription. CDKs require the presence of a regulatory cyclin subunit to acquire full activity [62]. To date, 20 CDKs and more than 30 cyclins have been identified [63]. Of note, the typical cell cycle-associated CDKs, like CDK1-5 and others, are characterized by multiple cyclin-binding, while transcriptional CDKs, like CDK7–9, are single cyclin-binding. The term “cyclin” was originally defined as based on the characteristic cyclic pattern of accumulation and degradation (reviewed by [62,64]). Cyclins of types A, B, C, D, and E exhibit this oscillating expression [65]. Recent evidence indicated that these oscillating cyclins can also regulate DNA damage repair and apoptosis, in addition to their known role in cell-cycle regulation [66]. Transcriptional cyclins, including T, K, L, Q, C, and H, associate with their corresponding transcriptional CDKs in order to regulate RNA polymerase II (RNAPII) activity and specificity as well as other cellular processes involved in gene transcription [67,68]. Interestingly, some cyclins have CDK-independent functions. For instance, cyclin D1 can act as an oncogene by modulating the transcriptional activity of estrogen receptors [69]. A common feature of all cyclins is the “cyclin box”, which is an approx. 100-amino-acid domain that forms a stack of five α-helices and is crucial for binding and activating CDKs [63]. It is especially noteworthy that intense research has been conducted on the virus-supportive role of CDKs in infected cells [70]. In this context, it appears striking that viruses, in particular herpesviruses, evolved a number of mechanistically different modes to interact, interfere, or cross-talk with components of the host CDK–cyclin machinery [40,71,72,73]. Such interactions are highly effective, since CDKs are tightly regulated through various mechanisms, including oscillation of cyclin expression, phosphorylation, or dephosphorylation of CDK–cyclin complexes, CDK inhibitors, and the subcellular localization of CDKs [62,74,75]. CDKs are defined by a catalytic core, which comprises the ATP-binding pocket, a PSTAIRE-like cyclin-binding domain in the C-lobe, and an activating T-loop motif [76]. The activation process of CDKs can be exemplified by CDK2–cyclin A. In its inactive form, the T-loop blocks the active site and the activation segment in the C-lobe is misoriented, which is responsible for binding the Ser/Thr phospho-acceptor region of substrates. Cyclin A binding induces hydrophobic interactions between the PSTAIRE-containing C-helix and a specific cyclin helix, resulting in a rotation that partially activates the kinase. The full kinase activity is achieved after the phosphorylation of the T-loop at Thr160 by the CDK-activating kinase (CAK) [62,77].

The CDK-activating kinase (CAK) complex is of particular interest, because the CAK complex itself is a CDK, consisting of CDK7, cyclin H, and MAT1, a RING-finger binding protein [78]. This heterotrimeric complex activates cell cycle CDKs through T-loop phosphorylation, specifically CDKs 1, 2, and 4/6, as well as transcriptional CDKs such as CDK9 [79,80]. Additionally, the CAK plays a direct role in regulating transcription as part of the general transcription factor TFIIH. CDK7 kinase activity requires cyclin H binding, whereas MAT1 stabilizes the CAK complex and anchors it to TFIIH [68]. MAT1 binding results in greater substrate specificity of CDK7 for RNAPII compared to CDK7–cyclin H, which preferentially phosphorylates CDK2 [81,82,83]. Although T-loop phosphorylation is not obligatory for CDK7 activity, phosphorylation at Ser164 and Thr170 enhances both its kinase activity and affinity for cyclin H. Furthermore, CDK7 activity is directed toward RNAPII instead of CDK2 through CDK7 phosphorylation at Thr170. The CAK complex can also undergo autophosphorylation, leading to reduced activity of CDK7–cyclin H but no observable impact on the ternary CDK7–cyclin H–Mat1 complex [82].

### 2.2. Herpesviral Protein Kinases as a Potential Novel Group of Antiviral Drug Targets

Considering the fact that all herpesviruses encode one or two own protein kinases in the form of the UL- and US-types of HvPKs, intense functional investigations have been performed for α-, β-, and γ-herpesvirus. Hereby, relevant roles of HvPKs in various aspects of viral replication were identified. Although the question of an essential role in viral replication was challenged through the reconstitution and analysis of viral deletion mutants, the support of a high replication efficiency through HvPKs was clearly demonstrated. Some examples illustrated that HvPK gene deletion could be tolerated to maintain a basic level of viral replication, e.g., for the HSV-2 US3 kinase. However, the identification of numerous phosphorylated substrates and multifaceted regulatory tasks clearly substantiated the notion that each of these HvPKs has importance for viral replication. Many relevant studies have been performed on the pUL97 kinase of HCMV, which initially had been identified as an enzyme that activates the nucleoside analog GCV through a step of initial nucleoside monophosphorylation [84,85]. Essentially, pUL97 is a tegument protein that is packaged into virions and is expressed with early-late kinetics. It represents the largest herpesviral kinase, which is expressed as three isoforms M1, M74, and M157, comprising approx. 100, 80, and 70 kDa, respectively. The largest, dominant isoform M1 (707 amino acids) mainly localizes in the nucleus due to two distinct nuclear localization signals (NLS) present in its poorly structured N-terminus [36,86]. Lysine 355 (K355) is essential for kinase activity; deletion or mutation of this position renders the kinase inactive.

The HCMV-encoded protein kinase pUL97 is a major target for antiviral therapy utilizing two types of inhibitors. Firstly, nucleoside analogs that require phosphorylation by pUL97 to become effective, and, secondly, direct-acting inhibitors of the pUL97 kinase activity. Ganciclovir (GCV), and its valine-esterified prodrug valganciclovir (VGCV), having been approved since 1989, are still widely used as the first-line treatment for severe HCMV infections, especially in immunocompromised patients [87]. Years after FDA approval, vCDK/pUL97 was discovered to be responsible for the activating phosphorylation of GCV [85]. GCV resistance mutations in ORF-UL97 rapidly emerged after approval clustering at codons coding for amino acids 460, 520, and 590–607. These mutations result in a 5- to 15-fold increase in the half-maximal effective concentration (EC_50_) of GCV-mediated antiviral activity (i.e., through a mechanism that is indirect, in that mutations in ORF-UL97 reduce phosphorylation-mediated prodrug conversion of GCV [46]). In contrast to the mode of action of nucleoside analogs, the benzimidazole L-riboside maribavir (MBV) represents a direct, competitive inhibitor of the ATP binding site of pUL97 with high antiviral efficacy [43]. MBV has been investigated since the 1990s, but two failed phase III clinical studies delayed its approval [88,89]. This initial clinical failure of the drug may have been due to suboptimal dosing decisions and to the fact that the targeted kinase, pUL97, is not absolutely essential for viral replication [90], yet it plays a number of important regulatory roles in the various replicative stages. Thus, a restart of clinical characterization of MBV in several modified phase II and III trials finally led to the achievement of final endpoints. The approval of MBV by the FDA in 2021 (Livtencity^®^) for the treatment of post-transplant HCMV infection resistant to standard antiviral therapy marked a breakthrough in antiviral therapy [2]. With MBV/Livtencity^®^, for the first time, a direct acting viral kinase inhibitor has been approved in the entire field of antiviral therapy. However, resistance mutations again emerged rapidly, some even conferring resistance to both GCV and MBV treatments (i.e., ORF-UL97 mutations that reduce the phosphorylation of GCV or the activity of MBV, respectively), highlighting the need for additional therapeutic options [46,91].

The mechanistic role displayed by pUL97 in the course of HMCV replication is primarily directed to regulation of the viral nucleocytoplasmic capsid egress through phosphorylation of the nuclear lamina, i.e., an impairment of pUL97 functionality shows the main phenotype of nuclear capsid retention. A second important mechanistic aspect of pUL97 concerns the G1/S phase checkpoint transition, which is mediated by pUL97 phosphorylation of the retinoblastoma (Rb) checkpoint regulator [61,92,93]. Consequently, HCMV mutants with a deletion of the UL97 gene, or those expressing pUL97 lacking its kinase activity, display severe replication defects [44,45,90,94,95,96]. Due to these different modes of action and targeting, MBV demonstrates antiviral activity against most viral mutants resistant to DNA polymerase inhibitors GCV, cidofovir (CDV), or foscarnet (FOS) [43,97,98]. Interestingly, as MBV inhibits the viral kinase necessary for activation of GCV, the two drugs act antagonistically and cannot be administered in combination [99]. MBV is exclusively administered orally, with subsequent absorption of approx. 30%-40%, and has been shown to be safe and well-tolerated, with common side effects including taste disturbances and headache [100].

## 3. The Viral Cyclin-Dependent Kinase Ortholog (vCDK)

### 3.1. Structural Similarity between Herpesviral and Host Kinases of the CDK Group

Most notably, the HCMV kinase pUL97 interacts with human cyclins T1, H, and B1 [101,102,103,104]. Two crucial regions in pUL97 have been identified for cyclin interaction. Interface 2 (IF2) is a short-sequence stretch within the unstructured N-terminus, consisting of amino acids 231–280. Interface 1 (IF1) is a larger contact region located in the C-terminus globular kinase domain, consisting of amino acids 329–634 [40]. Deletion of IF2 resulted in a complete loss of both pUL97 oligomerization and interaction with cyclin T1 and H [47,101,102]. On the other hand, IF1 is believed to have a more general role in cyclin binding. This means that IF2 represents the primary interface for the interaction with cyclins T1 and H, while IF1 exerts an accessory function that reinforces the binding affinity.

**Figure 2:**
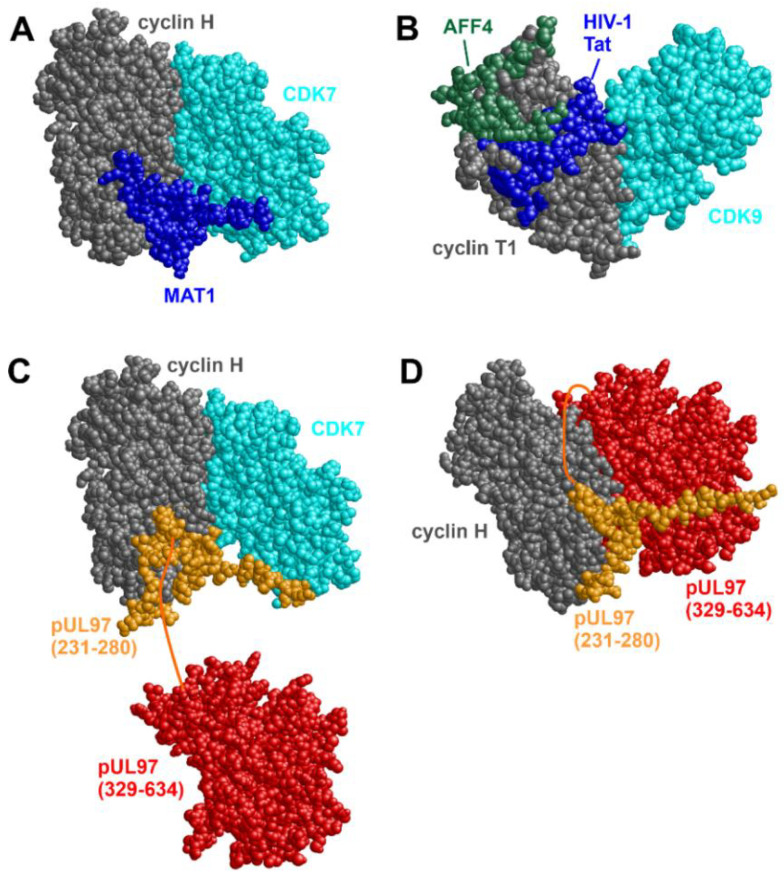
Cyclin interaction sites and model of pUL97–cyclin H complex. (**A**) Structure of the ternary CDK7–cyclin H–MAT1 complex (PDB code: 7B5O [106]). (**B**) Structure of the quaternary CDK9–cyclin T–Tat–AFF4 complex (PDB code: 4OR5 [107]). (**C**) Model of a ternary pUL97–cyclin H–CDK7 complex, in which pUL97 is attached to cyclin H exclusively through IF2 formed by the 231–280 sequence stretch. The pUL97 kinase domain (residues 329–634, marked in red) is connected to the complex by a nonstructured, flexible linker (residues 281–328, indicated as dark orange connecting line). (**D**) Model of a pUL97–cyclin H complex, in which pUL97 interacts with cyclin H both through IF2 (orange), pUL97(231–280), and the globular kinase domain IF1 (red), pUL97(329–634), thereby displacing CDK7. Panels (**C**,**D**) adapted from [105].

The observation of two distinct cyclin-binding regions in pUL97 raised the question about structural details of this interaction. First insight into the general properties of cyclin recognition could be gained from the experimental structures of cyclin H and T1 in complex with various cellular and viral interaction partners (Figure 2A,B). Both cyclins use a similar surface patch for binding the kinase domains of their respective cellular CDKs (CDK7 for cyclin H, CDK9 for cyclin T1). In addition to this canonical interface, the cyclins use various additional interfaces to bind short-sequence stretches of additional cellular (e.g., MAT1, AFF4) or viral regulatory proteins (e.g., human immunodeficiency virus type 1 Tat). Thus, cyclin H and cyclin T1 exhibit multiple binding sites that enable the formation of ternary or even higher-order molecular complexes. For HCMV vCDK/pUL97 and its interaction with cyclins, no experimental 3D-structure is available to date. A recent study [105] used an AI-/AlphaFold-based approach to predict the mode of interaction between pUL97 and cyclin H. This modeling approach suggested that the short and functionally essential cyclin binding patch (IF2) of pUL97(231–280) uses a similar interface region as MAT1 for targeting cyclin H (Figure 2C). This binding site does not overlap with the interface for kinase binding; therefore, the pUL97(231–280)–cyclin H complex should still allow for a simultaneous binding of the CDK7 kinase domain (Figure 2C). Alternatively, the kinase domain of pUL97 might replace CDK7 by forming additional interactions with cyclin H via IF1 (Figure 2D). This mode of interaction would explain the accessory function of IF1 by increasing pUL97–cyclin H binding affinity. In summary, molecular modeling strongly supports the previously suggested mode of interaction between pUL97 and cyclin H via the two distinct interfaces, IF1 and IF2, which allows the formation of both binary pUL97–cyclin H complexes and ternary pUL97–cyclin H–CDK7 complexes [105]. Modeling of the pUL97–cyclin T1 interaction suggests that complex formation relies on a simultaneous use of IF1 and IF2, thereby allowing for the formation of higher-order molecular complexes [102].

On the basis of the structural and functional data, the HCMV kinase pUL97, and other kinases of β- and γ-herpesviruses, have been considered as viral CDK orthologs and were termed vCDKs [37]. Of note, similar to their cellular counterparts, vCDKs phosphorylate substrates including Rb, lamin A/C, cyclin B1, among others, and can overcome cellular arrest in CDK-depleted yeast complementation assays [37,57]. HCMV pUL97, in particular, shares additional substrates with CDKs, including pUL69 [108], the pUL50–pUL53 nuclear egress complex [109,110], the C-terminal domain (CTD) of RNAPII [111], SAMHD1 [112], and EF-1δ [113]. Although the sequence identity with CDKs is relatively low, alignment of the pUL97 C-terminal kinase domain with CDK2 suggests that functional residues in the ATP binding site and the catalytic center are conserved [37,114]. Unlike classic CDKs, pUL97 does not appear to require phosphorylation of its predicted T-loop. Furthermore, CAK inhibition does not impact the activity of pUL97 [57,104]. Another difference between pUL97 and CDKs is that pUL97 lacks the PSTAIRE helix, which is typically the main mediator for cyclin binding. Cyclin binding in pUL97 is more likely mediated by electrostatic interactions between the binding regions of pUL97 and cyclins, as could be demonstrated in the case of CDK1–cyclin B1. Here, a negatively charged motif in cyclin B1 interacts with a positively charged region of CDK1 independent of the PSTAIRE motif [115].

### 3.2. Functional Similarities and CDK-Like Activities of Herpesviral Protein Kinases

**Figure 3:**
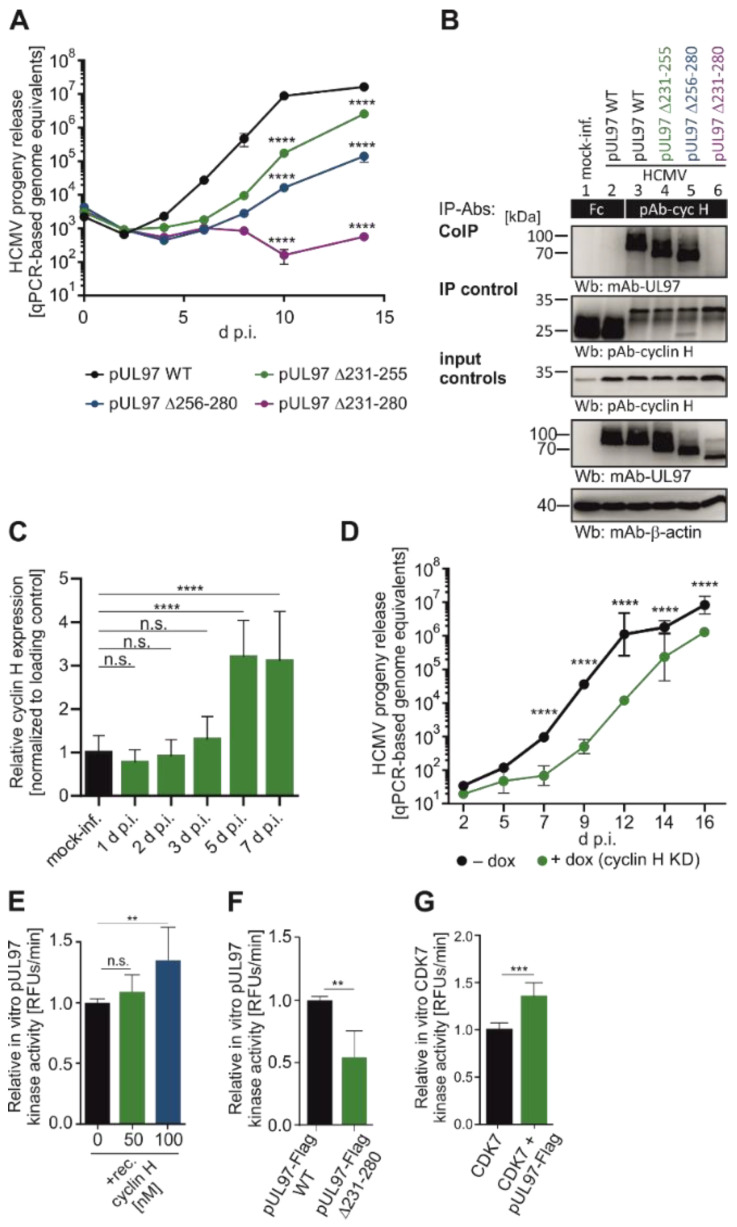
Importance of pUL97–cyclin H interaction for viral replication and kinase activity. (**A**) HFFs were infected with HCMV (recombinant TB40-IE2-YFP) WT or pUL97 IF2 deletion mutants. Viral genome equivalents of released virions were determined by qPCR. (**B**) HFFs infected with HCMV (recombinant TB40-IE2-YFP) WT or pUL97 IF2 deletion mutants at MOI 0.01 were harvested and lysed 4 d.p.i., before cyclin H was immunoprecipitated and the coimmunoprecipitation of pUL97 was analyzed by Wb. (**C**) Cyclin H protein quantities were analyzed from TEV-1 cells infected with HCMV Merlin by densitometric quantitation of Wbs at specified time points. (**D**) HFFs with doxycycline-inducible cyclin H knockdown were infected with HCMV AD169, and viral genome equivalents were determined by qPCR (for various cellular controls of this approach, see [102,105,138]). (**E**) 293T cells transfected with pUL97-Flag or empty vector were analyzed for kinase activity using a qSox-IVKA with increasing concentrations of recombinant cyclin H. (**F**) pUL97-Flag WT and Δ231–280 mutant were analyzed for kinase activity using a qSox-IVKA. Values were normalized to pUL97-Flag WT. (**G**) 293T cells transfected with pUL97-Flag or empty vector, and pUL97-Flag and/or CDK7 were immunoprecipitated and analyzed by a qSox-IVKA. Kinase signals were normalized to CDK7. ****, *p* < 0.0001; ***, *p* < 0.001; **, *p* < 0.01; n.s., not significant. For detailed methodology, see [104,139].

The HvUL protein kinase pUL97, when overexpressed in *Saccharomyces cerevisiae*, showed the ability to rescue a G1/S cell cycle defect of a yeast mutant that lacked CDK function [57]. This yeast complementation assay clearly illustrated the potency of pUL97 to substitute for cellular CDKs, thus characterizing this HvUL protein kinase as a CDK ortholog. A further report addressed the question whether the CDK-like activities of pUL97 were also shared by other members of the HvUL group of protein kinases. As an important finding, it was described that the ability to phosphorylate Rb and lamin A, and to disrupt the nuclear lamina, was shared by HvUL protein kinases from the β- and γ-herpesvirus subfamilies, but not by their α-herpesvirus homologs [37]. Another study [116], however, indicated that morphological alteration of lamin A can be induced by the α-herpesviral UL13 kinase, possibly in a cell-type-specific manner. These findings strongly support the idea that β- and γ-HvUL protein kinases share a conserved CDK-like function and may generally be considered as vCDKs (Figure 3 [37,57,58,108]). Further detailed functional analysis of the various HvPKs may substantiate this suggestion.

### 3.3. Identical Substrate Proteins and Host Interactors Shared by Host CDKs and vCDKs

It appears striking for the well-studied example of the HCMV vCDK/pUL97 that several identical substrate proteins are shared with host CDKs (see Figure 1). These substrates may thus underly a mode of dual phosphorylation through two different types of kinases, albeit closely related, in virus-infected cells. Very interesting findings have been collected in this aspect for Rb, RNAP II, SAMHD1, cyclin B1, and viral proteins like pUL69 [37,40,117]. These proteins, collectively recognized as pUL97-specific substrates, are also phosphorylated by host CDK–cyclin complexes, and thus may underlie dual phosphorylation upon HCMV infection. Although the latter point has not been proven in all details as well as in its functional significance, for some of these substrates the specific target phosphosites of pUL97 have been identified. Notably, some of these phosphosites are even identical with those recognized by CDKs. In particular for host proteins, this dual mode of site-specific phosphorylation has been validated, such as for lamins A and C (e.g., S22), retinoblastoma protein Rb (e.g., T356, T373, S608, S612, S780, S788, S795, S807, S811, T821, and T826) as well as SAMHD1 (e.g., T592) [37,112,118,119,120,121,122]. For other host proteins, subject to pUL97-specific phosphorylation, such as the intrinsic immune factor IFI16, less information has been provided in terms of phosphosites [123].

One specifically interesting aspect has been provided by the study of the regulatory role of host prolyl cis-trans isomerase Pin1 in HCMV infection. The virus-supportive function of the host factor Pin1 is very relevant in this context, because the activities of Pin1 and the HCMV kinase pUL97 are closely interconnected. The general activity of Pin1, as a proline-directed cis-trans isomerase, is to convert substrate proteins in terms of conformational changes. Specifically, during the identification of lamin A/C-specific phosphorylation by the viral kinase vCDK7/pUL97 in HCMV-infected cells, the role of Pin1 was postulated to be involved in lamin A/C cis-trans isomerization at pUL97-phosphorylated sites, such as pSer22 [122,124]. Notably, the Pin1 isomerase is a crucial regulatory protein that facilitates the cis-trans isomerization of phosphorylated serine/threonine-proline motifs in a number of cellular proteins [125]. Moreover, several studies have demonstrated that Pin1 plays a pivotal role in the regulation of virus replication. During infection, Pin1 may influence the progression of viral infection by regulating the host or viral protein functions. In the context of HCMV, Pin1 facilitates viral nuclear capsid egress through the locally focused nuclear lamina disassembly, resulting in lamina-depleted areas [125]. It has also been reported that Pin1 interacts with viral pUL44 and pUL69, probably both in a phosphorylation-dependent manner [126,127]. Likewise, involvement of Pin1 is also linked to several other herpesviruses, such as Epstein-Barr virus (EBV) and Kaposi’s sarcoma-associated herpesvirus (KSHV). In EBV, the induction of the lytic replicative cycle leads to the amplification of the EBV genome by 100 to 1000-fold. Pin1 specifically binds EBV DNA polymerase catalytic subunit, BALF5, and efficiently increases viral DNA replication [128]. It is speculated that Pin1 modulates the conformation of BALF5, which may lead to this enhanced efficiency. Interestingly, in KSHV infection, the viral transactivator Rta functions as a lytic switch protein and initiates the productive reactivation of KSHV. Rta is thought to serve as a substrate of Pin1-mediated isomerization, and this in turn can promote the reactivated lytic cycle, while Pin1 may likewise exert inhibitory effects in virus maturation. Thus, Pin1 may have a bidirectional role in KSHV infection [129]. In addition to herpesviruses, Pin1 substrates have also been recognized for other viruses. Tax protein of human T-cell leukemia virus type 1 (HTLV-1) [130], NS5A and NS5B proteins of hepatitis C virus (HCV) [131], hepatitis B virus (HBV) core and HBX proteins [132,133], N protein of SARS-CoV-2 [134], as well as HIV-1 capsid and integrase, were described [135,136,137]. Therefore, Pin1 appears to influence viral replication in several mechanistic ways.

### 3.4. Cyclin-Binding Properties of the Herpesviral CDK-Like Kinases

A specifically remarkable finding was the first demonstration of interaction of a UL-type HvPK with host cyclins, namely by HCMV pUL97. As far as the regulatory potential of the pUL97 kinase was concerned, its interactive property to form complexes with the cyclin types H, T1, and B1 has raised questions for a long time. Only recently, a combination between analyses of viral mutants and cyclin knock-down (KD) in host cells, pointed to the fact that human cyclin H possesses main functional relevance for supporting the efficiency of lytic HCMV replication [102,104,105,138,139]. In particular, the replication efficiency of tree viral partial deletion mutants in IF2 of pUL97 (i.e., HCMV AD169-GFP Δ231–255, Δ256–280, and Δ231–280) was analyzed by measuring the quantity of viral genome equivalents in the supernatant (Figure 3A) by qPCR. Deletion mutant Δ231–280 displayed a strong replication impairment, i.e., reduction of released genome copies, with no detectable increase of HCMV genome equivalents in the supernatant. The smaller deletion mutants Δ231–255 and Δ256–280 demonstrated an intermediate phenotype, resulting in a less severe replicative impairment. Then, the recombinant HCMVs were utilized to examine the binding capability of the pUL97 mutants to human cyclin H. Endogenous cyclin H was immunoprecipitated, and the CoIP of pUL97 was analyzed by SDS-PAGE and Western blot (Wb) analysis (Figure 3B). Crucially, HCMV pUL97 Δ231–280 lost its interaction with cyclin H (Figure 3B, upper panel, lane 6), whereas Δ231–255 and Δ256–280 mutants did not exhibit this phenotype and were comparable to the WT pUL97 interaction for cyclin H (lanes 4–5). Taken together, the largest deletion showed the most pronounced impairment regarding cyclin H binding and replicative fitness, while the two smaller deletions were found to be intermediate. A clear increase in the levels of cyclin H protein was observed upon infection with HCMV AD169-GFP (Figure 3B, lanes 2–6). To further explore this discovery, human first-trimester extravillous trophoblast cells (TEV-1) were infected with the clinically relevant HCMV strain Merlin. A densitometric analysis of Wb bands indicated a significant, time-dependent upregulation with particularly high levels observed at later time points between 3 to 7 d p.i. (Figure 3C). In the next step, a primary human foreskin fibroblast (HFF) population with a stably transduced doxycycline (dox) inducible cyclin H-specific short hairpin RNA (shRNA) was generated and then used for qPCR-based infection kinetics (Figure 3D). A significant 100-fold reduction in the release of viral genome equivalents could be detected when comparing dox-induced cyclin H KD cells to uninduced cells. At this point, the question remained open whether the strong decrease in viral replication under cyclin H KD conditions is directly related to the interaction between pUL97 and cyclin H or is indirectly linked to the deregulation of CDK7 caused by depletion of cyclin H. Previous studies have demonstrated that inhibition of CDK7 strongly inhibits the replication of HCMV, rendering it a promising target for antiviral therapy [140,141]. To rule out the possibility that the growth defect caused by cyclin H KD was only due to indirect effects mediated by CDK7, the direct impact of cyclin H on pUL97-specific kinase activity was investigated using a recently developed, fluorescence-based in vitro kinase assay (qSox-IVKA [138]), in various approaches (Figure 3E,F). Transiently expressed, immunoprecipitated pUL97-Flag was supplemented with recombinant cyclin H for the qSox-IVKA reaction (Figure 3E). The addition of 100 nM recombinant cyclin H increased the in vitro kinase activity of pUL97 by 31.5%. It should be noted that the impact of exogenously added cyclin H on pUL97 kinase activity was expected to be modest, since whole-cell lysate containing endogenous cyclin H was used for immunoprecipitation of pUL97. Another qSox-IVKA was conducted to determine whether a pUL97 mutant lacking the IF2 region for cyclin H interaction (amino acids 231–280) exhibited reduced kinase activity compared to full-length pUL97 (Figure 3F). To this end, transiently expressed pUL97 ∆231–280-Flag and pUL97-Flag were prepared. To compare the pUL97 kinase activity of two distinct samples, the quantities of the immunoprecipitated pUL97-Flag samples were determined and normalized by Wb analysis and densitometry. Finally, the activity values of pUL97-Flag Δ231–280 were normalized to the values derived from the pUL97-Flag WT. Strikingly, the pUL97 kinase activity of the deletion mutant pUL97 ∆231–280 was reduced to 53% compared to full-length pUL97.

**Figure 4:**
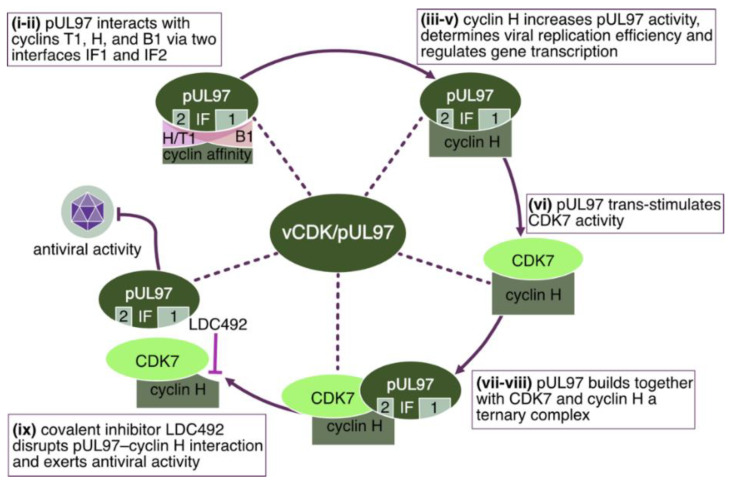
Scheme summarizing the complex regulatory interaction between vCDK/pUL97, cyclin H, and CDK7. Details of regulatory protein complex formation as well as the aspired drug-targeting functions are depicted. A specific focus is given to the options of developing mechanistically novel antiviral drugs directed to this complex. Detailed explanations are given in Section 3.4, Section 5.3, Section 6 and Section 7; for further details, see [105].

As another aspect of this kinase-interactive regulation, it was investigated whether pUL97, which can form ternary complexes with cyclin H and CDK7 [104], could directly stimulate CDK7 kinase activity (Figure 3G). To address this question, the two kinases pUL97 and CDK7 were immunoprecipitated separately or together. For the following qSox-IVKA, a CDK7-specific Sox peptide (barely recognized by pUL97) was used as a kinase sensor. The kinase activity of CDK7 and pUL97 single immunoprecipitates was measured. Then, the pUL97 single kinase activity was subtracted from the coimmunoprecipitated activity values to obtain the specific kinase activity of CDK7. Remarkably, the CDK7 activity observed in the samples transfected with pUL97 exhibited a significant increase of 35.1% compared to controls, suggesting a mode of trans-stimulation of CDK7 by the viral kinase pUL97. Taken together, the HCMV-encoded vCDK/pUL97 undergoes regulatory complex formation with both cyclin H and CDK7, which consequently supports viral replication efficiency (Figure 3) and represents a multifaceted targeting point for antiviral strategies (Figure 4).

## 4. Evidence for the Functional Significance of HvPKs in Herpesviral Replication

### 4.1. Functional Aspects of the Conserved (UL) and Nonconserved Gene Group (US) of HvPKs

It is generally agreed that the conserved group members of HvPKs are not absolutely required for viral replication in cell culture, but deletion mutants may show severe attenuation and viral defective phenotypes ([37] and references therein). They are expressed with early-late kinetics and incorporated into virions. The various functions, in which HvPKs are involved during viral replication, comprise tegument disassembly, modulation, or stimulation of gene expression and viral genomic replication, and to facilitate capsid nuclear egress through a phosphorylation-specific rearrangement of the nuclear lamina. Importantly, the pUL97 kinase HCMV was shown to directly phosphorylate the cellular retinoblastoma (Rb) tumor-suppressor protein both in vivo and in vitro, on residues that are normally targeted by the cellular CDKs that control cell cycle progression [57]. Significantly, experiments with a yeast-complementation assay demonstrated that pUL97 can functionally substitute for cellular CDK activity, thus indicating that pUL97 acts as a virus-encoded CDK-like kinase (vCDK). Moreover, it was shown that the HvPKs encoded by the β- and γ-herpesviruses are all capable of inducing Rb phosphorylation in vivo on residues that inactivate the cell cycle inhibitory and tumor-suppressor function of this protein. They can also induce lamin A phosphorylation and disrupt the nuclear lamina. Importantly, all the β- and γ-herpesviral PKs, with the exception of the HHV-8 (KSHV) ORF36 protein, displayed authentic CDK function in the yeast-complementation assay [37]. In contrast, the α-herpesviral PKs appear to be unable to phosphorylate Rb or lamin A, unable to efficiently disrupt the nuclear lamina, or complement a CDK activity in S. cerevisiae ([37] and references therein).

Studies on HSV mutants lacking the UL13 gene have provided contradictory data. Early research indicated that the UL13 kinase was not essential for viral replication in cell culture. However, later studies revealed that these mutants have impaired replication, dependent on the cell type, with reduced levels of the immediate early ICP0 protein and several late proteins such as UL26, UL26.5, UL38, UL41, and US11. Various viral and cellular targets of UL13 have been identified, but the biological significance of UL13-mediated phosphorylation remained uncertain. Recent findings provided evidence that UL13 plays a role in regulating nuclear egress, in particular as UL13 deletion causes abnormal localization of HSV egress factors UL31 and UL34. Additionally, UL13 is thought to aid in tegument dissociation and additional regulatory activities in viral replication ([41] and references therein, [142,143]).

The VZV ORF47 protein is analogous to HSV UL13 and is involved in autophos-phorylation and phosphorylation of several key proteins, including the immediate early transactivators IE62 and IE63, the early regulators ORF9 and ORF32, as well as the later-phase viral glycoprotein gE. The gE protein is crucial for VZV replication and relies on ORF47 phosphorylation for cell fusion and trafficking for virion assembly. Although ORF47 is not necessary for VZV replication in vitro, it is essential for infection in differentiated human T cells, skin xenografts in the SCID-hu model, and immature dendritic cells. ORF47 and ORF66 protein kinases activate the PI3/Akt/GSK-3α/β pathway, which is critical for viral infection. Cell culture-based in vitro studies of ORF47 showed that it preferentially phosphorylates serine residues using ATP and GTP. There is conflicting evidence on whether ORF47 is involved in nucleoside analog metabolism. Some studies suggest that ORF47 contributes to the phosphorylation of certain nucleoside analogs, while others find no involvement of ORF47 in this process. Further functional aspects are discussed ([41] and references therein, [144,145]).

The EBV BGLF4 protein kinase is expressed early during the lytic phase and is primarily found in the nuclei of infected cells. Its nuclear localization signal is at the C-terminus. Knocking down BGLF4 by RNAi results in nuclear retention of nucleocapsids, similar to HCMV pUL97 mutants. This KD also abolished BFLF2 expression, indicating a role of BGLF4 in nuclear egress. Transient expression of BGLF4 causes chromosome condensation, nuclear lamina disassembly, and stress fiber rearrangements, independent of DNA replication and CDK2 activity. BGLF4 interacts with condensin complexes, phosphorylates them, and stimulates topoisomerase II, which may aid chromosome condensation and viral DNA replication. Moreover, BGLF4 phosphorylates the MCM4-MCM6-MCM7 complex, inhibiting helicase activity and possibly blocking cellular DNA replication during lytic infection. BGLF4 is a tegument protein that dissociates in a phosphorylation-dependent manner. While it phosphorylates various targets, its biological significance, aside from viral nuclear egress, is mostly hypothetical. BGLF4 has been expressed in insect cells, where it autophosphorylates, phosphorylates histone and myelin basic protein, and uses ATP and GTP. Unlike β-herpesviruses, γ-herpesviruses like EBV encode thymidine kinase (TK), which confers sensitivity to several antiviral drugs. BGLF4 confers sensitivity to GCV, and BGLF4 has been shown to catalyze GCV prodrug activation in vitro, most probably through direct GCV phosphorylation ([41,146] and references therein). In addition, the contribution of BGLF4 in the regulation of intrinsic immunity and cell cycle progression has been analyzed [147,148].

Concerning the nonconserved gene group (US) of HvPKs, US3 orthologs in HSV-1, HSV-2, and VZV were characterized in the late 1980s and were likewise found to resemble known human or other eukaryotic protein kinases. Using antisera and kinase assays, HSV US3 was definitely confirmed as a viral protein possessing kinase activity. This kinase is conserved in α-herpesviruses, but absent in other herpesviruses. Recent studies revealed that HSV-1 US3 has more substrates than initially believed. Autophosphorylation at serine 147 influences US3 function and localization. While several potential US3 substrates have been proposed, few are confirmed as biologically significant. Studies of US3 mutant viruses show that although these mutants grow moderately well in cell cultures, they are significantly less virulent in animal models, establishing US3 as crucial for viral replication and pathogenicity. US3 is involved in various functions, some conserved among α-herpesviruses and others unique to specific viruses. US3 kinase activity is essential for most of these functions, particularly in nuclear egress. In cells infected with US3-null viruses, virions accumulate abnormally in the perinuclear space, suggesting a role of US3 in nuclear egress. This defect is not absolute, as extracellular virus titers are only mildly reduced without US3. Specifically, US3 phosphorylates lamin A/C and emerin, disrupting the nuclear lamina and facilitating nucleocapsid access to the inner nuclear membrane. US3-null infections alter the distribution of UL34 and UL31, crucial for nucleocapsid envelopment, from a continuous to an aggregated pattern. Also, US3 phosphorylates gB, a protein essential for membrane fusion during viral entry, potentially aiding the fusion of primary enveloped virions with the outer nuclear membrane. This function may not be conserved across all α-herpesviruses, as gB and gH do not appear at nuclear membranes in case of pseudorabiesvirus (PRV) infection. These findings underscore the pivotal role of the US3 kinase in viral replication and pathogenicity, especially through its regulatory activity in nuclear egress ([149] and references therein).

## 5. Chances, Challenges, and Current State of vCDK- and CDK-Specific Antiviral Drugs

### 5.1. Antiviral Validation Steps of the HCMV vCDK/pUL97

As exemplified in Section 4.1, HvPKs cover a relatively wide spectrum of viral and cellular substrate proteins, and thus exert multifunctional roles for viral replication in vitro and in vivo. Based on this knowledge, there has been a long-lasting debate whether these viral kinases might serve as suitable antiviral targets, to be attacked by virus-specific kinase inhibitors. A very clear answer has been given by the clinical approval of MBV as a therapeutically beneficial inhibitor of the HCMV vCDK/pUL97. This success has been considered a lighthouse in the implementation of kinase inhibitors into antiviral therapy (as a strategy originating from antitumor and antiinflammatory therapies). Even more, it has raised the question whether kinase inhibitors might open up further antiherpesviral drug development. However, the validation of the various HvPKs, for the purpose of antiviral drug targeting, still awaits scientific confirmation. In the case of HCMV, this has been achieved in several experimental levels, thus highlighting pUL97 as a highly interesting antiviral target.

First, genetic deletion approaches demonstrated the importance of pUL97 expression for a wild-type efficiency of high-level viral replication (see Section 2 and Section 4). Any defect in pUL97 expression substantially reduced HCMV replication and the production of infectious virus. Similar results were obtained with the use of pUL97-specific inhibitory small molecules. A number of pharmacological inhibitors of pUL97 have been described, derived from different chemical classes, which consistently confirmed a pronounced antiviral activity (reviewed in [40]). As a particularly interesting finding, a specifically strong anti-HCMV efficacy of MBV was demonstrated for non-dividing, quiescent cells [99]. This issue has been explained by the absence of certain CDK activities under these conditions, which might otherwise potentially provide functional complementation of the inhibited vCDK activity. The finding was then underlined by the KD experiments performed on host cyclins in HCMV-infected cells. It became evident that the KD of pUL97-supportive cyclin H in primary human fibroblasts represented a rate-limiting factor of low HCMV replication (see Section 3.4). Finally, a first indication of drug synergy between inhibitors of vCDK and host kinase activity (mTOR) was provided using cultured-cell models of HCMV infection. Specifically, the cotreatment of infection with MBV and rapamycin (sirolimus) led to a significant antiviral drug synergy [150]. Combined, these experimental approaches illustrated the successful validation of HCMV vCDK/pUL97 as a drug-accessible target, with possible consequences also for other antiherpesviral treatment options.

### 5.2. Discovery of Host CDKs as Very Potent Targets for Novel Anti-HCMV Strategies

**Figure 5:**
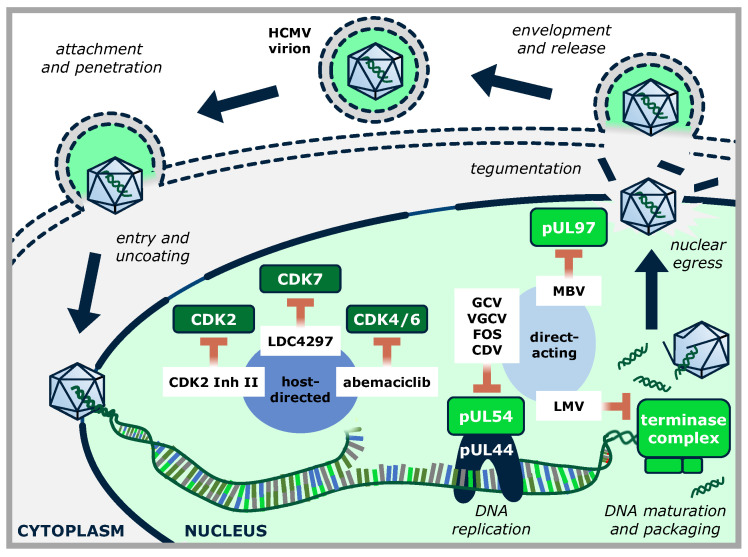
Schematic depiction of the HCMV replication cycle. Specific consideration is given to approved, direct-acting antivirals and their viral target proteins (light green boxes), as well as experimental host-directed antivirals and their cellular target proteins (dark green boxes). Steps of the viral replication cycle are given in italics. Details of the scheme are explained in the text. The primary data presented in this section, i.e., here appearing in a refined presentation style, have been published elsewhere [141,159].

**Figure 6:**
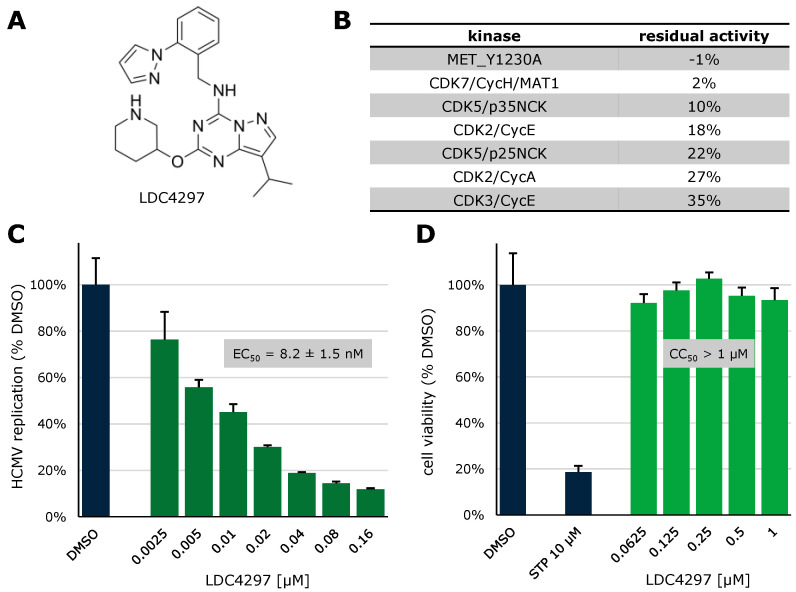
Characterization of the selective CDK7 inhibitor LDC4297 for anti-HCMV activity. (**A**) Structural formula of LDC4297. (**B**) Residual activity of top seven most inhibited cellular kinases under treatment with 100 nM LDC4297. Radiometric protein kinase assays were performed for 333 individual protein kinases [140]. Note the low degree of secondary inhibitory activity against kinases other than CDK7; the tumor-relevant mutant of MET kinase, Y1230A, has no relevance for a normal cellular background. (**C**) Antiviral profile of LDC4297 against HCMV AD169-GFP replication, at MOI of approx. 0.01, as determined by GFP-based replication assay for 7 days, using total lysates of infected HFFs. Data is shown as mean between two biological replicates with standard deviation (SD), normalized to solvent control DMSO (GFP fluorometric measurements in quadruplicate). Half-maximal effective dose (EC_50_) is given in the grey box as mean between two biological replicates ± SD. (**D**) Cytotoxicity profile of LDC4297 in HFFs as determined by Neutral Red uptake assay. Data is shown as mean between three biological replicates with SD, normalized to solvent control DMSO. Approximation of half-maximal cytotoxic dose (CC_50_) is given in the grey box. Staurosporine (STP) was used as a positive control.

Standard therapy of HCMV infection relies on a limited pool of antiviral medication, with six drugs approved for the prophylaxis or therapy of HCMV disease by the US Food and Drug Administration (FDA) (Figure 5). The gold standard of HCMV treatment and first-line drug is the acyclic nucleoside analog ganciclovir (GCV), as well as its oral prodrug valganciclovir (VGCV). After three phosphorylation steps by pUL97 and cellular kinases, GCV is able to compete with dGTP for the active site of the HCMV DNA polymerase pUL54, ultimately leading to chain termination [151,152,153]. The second line of HCMV treatment comprises the two drugs cidofovir (CDV) and foscarnet (FOS). As an analog of deoxycytidine monophosphate, CDV also targets viral DNA replication by pUL54, without the need for phosphorylation by pUL97; however, CDV is phosphorylated exclusively by cellular kinases [154]. During the elongation of newly synthesized viral DNA strands, pyrophosphate is cleaved from dNTPs by the viral polymerase complex. The second-line anti-HCMV drug FOS represents a structural analog to pyrophosphate and reversibly inhibits this process by competing for the pyrophosphate binding site of pUL54 [155]. In 2017, the treatment options for HCMV were expanded by approval of letermovir (LMV) for prophylaxis of HCMV infections in transplantation recipients [156,157]. LMV is an inhibitory small molecule of the pUL56 subunit of the HCMV terminase complex and constitutes the first approved compound of this inhibitor class [158]. The terminase complex cleaves the concatemeric HCMV DNA into monomeric genome length, and its inhibition prevents packaging of viral genomes into newly produced nucleocapsids [158]. The most recent drug approved for HCMV treatment is the benzimidazole L-riboside maribavir (MBV), an ATP-competitive inhibitor of the viral protein kinase pUL97 [43], FDA-approved in November 2021. Processes for which pUL97 fulfills an essential function include the nuclear egress of newly produced capsids, as well as the G1/S phase checkpoint transition, which is mediated by phosphorylation of the retinoblastoma (Rb) checkpoint regulator by pUL97 [61,92,93]. Our group investigated a number of selected experimental inhibitors targeting CDKs as putative candidates for next-generation antivirals against HCMV disease. An exemplary subset of these CDK inhibitors and their targets are shown in Figure 6.

In recent years, our group has focused on investigating host-directed compounds for antiviral use, with inhibitors of CDKs chief among them. The CDK7 inhibitor LDC4297 (Figure 6A) has previously been investigated by our group and has been shown to be highly CDK7-specific with weak secondary inhibition of CDK5 and CDK2 and virtually no activity against other kinases of the human kinome (Figure 6B, [140]). To confirm earlier findings of strong anti-HCMV activity for this compound, a GFP-based replication assay was conducted (Figure 6C), displaying a concentration-dependent decrease of HCMV replication with a half-maximal effective dose (EC_50_) of 8.2 nM, comparable with previous findings [159]. Cytotoxicity in relevant human foreskin fibroblasts (HFFs) was assessed using the Neutral Red uptake assay (Figure 6D), revealing no cytotoxic effects up to a concentration of 1 µM, resulting in a selectivity index (SI) of > 100.

**Figure 7:**
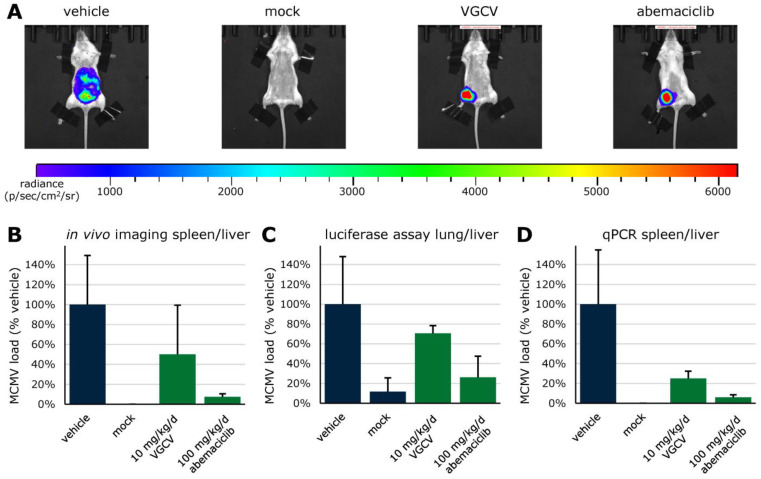
In vivo anti-MCMV activity of abemaciclib. (**A**) Exemplary images of in vivo luciferase imaging of four mice representative for respective treatment group. Color grading representing radiance is given in scale below. (**B**) Antiviral efficacy of abemaciclib treatment as assessed by in vivo luciferase imaging of spleen and liver. Data is shown as mean between analyzed organs with SD, normalized to vehicle control. (**C**) Antiviral efficacy of abemaciclib treatment as assessed by luciferase assay in lung and liver homogenates. Data are shown as mean between analyzed organs with SD, normalized to vehicle control. (**D**) Antiviral efficacy of abemaciclib treatment as assessed by viral genome-specific qPCR of DNA extracted from spleen and liver samples. Data are shown as mean between analyzed organs with SD, normalized to vehicle control. (Note, that the differences in (**B**–**D**) represent experimental tendencies that so far lacked statistical significance.

Along with LDC4297, additional inhibitors of a range of CDKs were analyzed for potential use as anti-HCMV active compounds via GFP-based replication assay and Neutral Red uptake assay (Table 1). Abemaciclib (ABE), a clinically approved CDK4/6 inhibitor, was further evaluated for its anti-CMV activity in vivo, using an immunocompetent Balb/c mouse model (Figure 7; EC_50_ of LDC4297 against MCMV was 0.07 ± 0.02, as determined in cultured fibroblasts). VGCV was used as a positive control at 10 mg/kg/d. Efficacy of this compound against MCMV replication has been previously demonstrated in the immune-deficient rag-/- mouse model [160]. Here, immunocompetent Balb/c mice were infected intraperitoneally with a luciferase-tagged MCMV reporter virus [161], which allowed for monitoring of viral replication by in vivo luciferase imaging (Figure 7A,B). Mice were daily treated with given concentrations of VGCV and ABE by oral administration and sacrificed on day 5 after infection. Luciferase assays of organ homogenates (Figure 7C) as well as qPCR on DNA extracted from organ samples (Figure 7D) were performed. Thus, treatment with 100 mg/kg/d of CDK4/6 inhibitor abemaciclib (i.e., within the dosage range of human clinical applications) reduced MCMV replication to 8%, 26%, and 6% in imaging, luciferase assay, and qPCR, respectively.

### 5.3. Synergistic Potential of Direct-Acting (vCDK) and Host-Directed (CDK) Kinase Inhibitors

**Figure 8:**
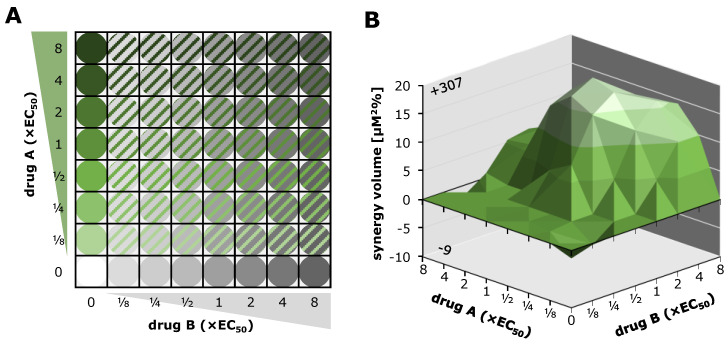
Loewe additivity fixed-dose dilution matrix and result visualization. (**A**) Dilution matrix employed for standard Loewe additivity fixed-dose assay. (**B**) CI value graph depicting exemplary synergistic drug interaction. Threshold between antagonistic and synergistic interaction (i.e., additive interaction) is marked by green dotted line. CI values at 50%, 75%, 90%, and 95% are given in bold. CI_wt_ is given in grey box.

The efficacy of combinatorial antiviral drug treatments in vitro was assessed using two established methods in a parallel approach in order to achieve highly reliable results. The Bliss independence checkerboard assay [162]) and the Loewe additivity fixed-dose assay [163]) were adapted to in vitro infection of HFFs with HCMV AD169-GFP. For the Bliss independence model, serial drug dilutions of two drugs A and B are combined in a checkerboard-like matrix (Figure 8A). The expected additive effect of a given combination is calculated utilizing the Bliss formula and then subtracted from the measured effect. The resulting synergy volume can be positive, zero, or negative, indicating synergistic, additive, and antagonistic interaction, respectively. Plotting the synergy volume for all combinations of the dilution matrix produces a synergy volume graph (Figure 8B), where strength of drug interaction corresponds to a larger volume above or below the 0 µM^2^% plane (classified drug interaction: below −100, strongly antagonistic; −100 to +50, additive; +50 to +100, moderately synergistic; above +100 strongly synergistic). The combination of MBV + GCV has been shown to exhibit strong antagonism before [159] and was therefore employed as a control. Combinations of an array of approved and experimental anti-HCMV compounds were investigated (Table 2). The tabular overview clearly illustrates that a number of kinase inhibitor combinations, especially pairs of direct-acting antivirals (DAAs) and host-directed antivirals (HDAs), exerted highly interesting antiviral profiles and statistically synergistic drug interactions (Table 2).

Please provide additional methods, including virus used, MOI, cells, time of analysis, nature of the readout.

**Figure 9:**
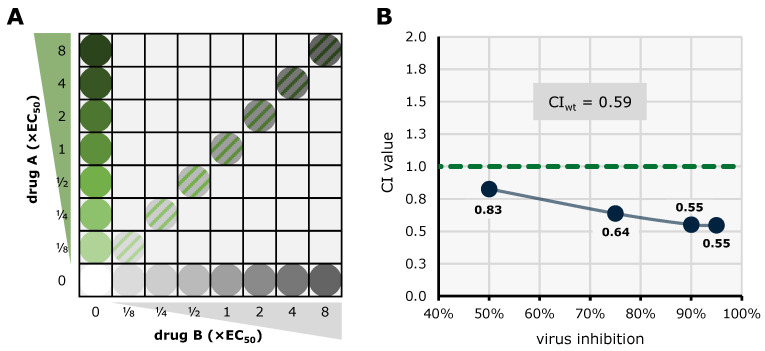
Loewe additivity fixed-dose dilution matrix and result visualization. (**A**) Dilution matrix employed for standard Loewe additivity fixed-dose assay. (**B**) CI value graph depicting exemplary synergistic interaction of two arbitrary drugs (hypothetical data derived from another context [159]). Threshold between antagonistic and synergistic interaction (i.e., additive interaction) is marked by a green dotted line. CI values at 50%, 75%, 90%, and 95% are given in bold. CI_wt_ is given in grey box.

As a second, confirmatory approach to investigate drug interactions, the Loewe additivity fixed-dose assay was employed. Here, a lower number of drug concentration pairs are analyzed, due to the ratio of drug A and B remaining constant (Figure 9A). From the dilution series of drug A, drug B and the combination AB, median effect plots are generated and compared at four points of efficacy (50%, 75%, 90%, and 95%) using the Loewe formula to calculate a combination index (CI) for each point; CI values below 1 and above 1 indicate synergism and antagonism, respectively (Figure 9B). From the CI values at 50%, 75%, 90%, and 95% efficacy, a weighted CI (CI_wt_) can be calculated (classified drug interaction: <0.1–0.3, strongly synergistic; 0.3–0.7, synergistic; 0.7–0.85, moderately synergistic; 0.85–0.9, slightly synergistic; 0.90–1.10, additive; 1.10–1.20, slightly antagonistic; 1.20–1.45, moderately antagonistic; 1.45–3.3, antagonistic; 3.3–>10, strongly antagonistic [164]). Results of the investigation of combinations of a broad range of approved and experimental anti-HCMV compounds can be seen in Table 3. Importantly, these results obtained with the Loewe additivity and Bliss checkerboard assays were strongly consistent with one another, in particular as far as the synergy between vCDK and CDK inhibitors is concerned.

**Figure 10:**
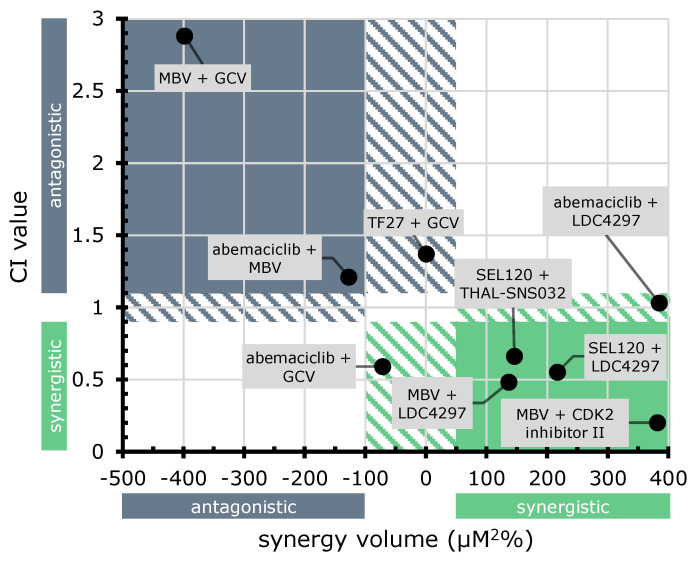
Comparative alignment of Bliss independence checkerboard and Loewe additivity fixed-dose results. X-axis value indicates mean synergy volume sum across checkerboard assays. Y-axis value indicates mean CI_wt_ across fixed-dose assays. Colored bars represent ranges of antagonistic (blue), and synergistic (green) values for each method. Solid blue/green fields within the chart indicate overlapping antagonistic/synergistic range of both approaches, respectively; dashed blue/green areas designate antagonistic or synergistic ranges in one method with additive values in the other method.

In order to visualize the combined results of Loewe additivity and Bliss checkerboard assays, CI values and synergy volumes were plotted against each other (Figure 10). This two-dimensional representation allows for visual assessment of synergistic (green) and antagonistic (blue) results in both analysis systems, as well as outcomes in intermediate areas (dashed green/blue). Two compound combinations exhibited antagonism in both approaches (MBV + GCV, abemaciclib + MBV); three combinations displayed results with one method indicating additive interaction, the other one antagonistic (TF27 + GCV) or synergistic (abemaciclib + GCV, abemaciclib + LDC4297). Concerning the question, which conclusions may be inferred from the synergy/antagonism results, it should be emphasized that these sorts of drug interactions are complex (including the regulatory interplay between targets, parameters of cellular uptake, stability, and secondary ligands), and may not easily be interpreted, even in cases in which the targets of inhibitors are known. Thus, more defined mechanistic analyses are required to address and explain these antiviral activities of CDK inhibitors [139]. Notably, synergistic interaction in both approaches was determined for four compound combinations of CDK + vCDK inhibitors (SEL120 + THAL-SNS032, SEL120 + LDC4297, MBV + CDK2 inhibitor II, and MBV + LDC4297).

**Figure 11:**
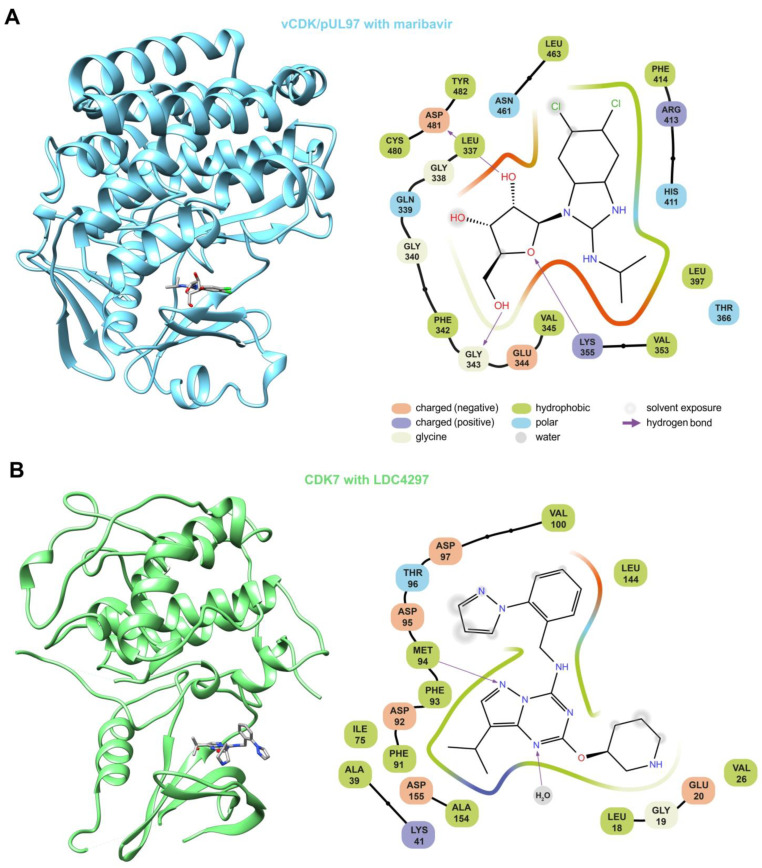
Structural depiction of drug-exposed contact amino acids. The comparative modeling setting illustrates the contact residues of vCDK/pUL97 (modeled with AlphaFold) and CDK7 (PDB ID: 8P7L [168]) that are involved in drug binding of MBV or LDC4297, respectively. (**A**) Prediction of the vCDK/pUL97 kinase domain and MBV docking to its ATP-binding pocket; and in comparison (**B**), prediction of the CDK7 kinase domain and LDC4297 docking to its ATP-binding pocket.

On the basis of this highly attractive option, to utilize synergistic drug combinations for putative future antiviral drug development, the question about the molecular binding modes of the aforementioned kinase inhibitors was addressed. To this end, a specific focus was directed to the pronounced synergy between CDK7 and vCDK/pUL97 inhibitors, as also substantiated by the physical and functional interplay between these two kinases in HCMV-infected cells. Figure 11 illustrates at the protein structure level how the two synergistic drugs maribavir (MBV, directed against viral pUL97) and LDC4297 (directed against host CDK7) interact with their kinases. MBV is a benzimidazole riboside, which competitively inhibits the ATP-binding pocket of vCDK/pUL97. Unfortunately, up to now there is no experimentally resolved protein structure of pUL97. In order to develop an idea of how MBV interacts with pUL97 in the ATP-binding pocket, the structure-prediction methods and docking had to be used at this point (as previously shown by us [165] and others [166,167]). For this review, we used AlphaFold to obtain optimized structure predictions of the kinase domain, and subsequently MBV was docked into the ATP-binding pocket (Figure 11A). In comparison, LDC4297, as a pyrazolotriazine-class compound, is a high-affinity and highly selective reversible inhibitor of CDK7. The complex structure of CDK7 with bound LDC4297 was recently determined by using high-resolution cryo-EM (PDB ID: 8P7L [168]; Figure 11B) and also by using X-ray diffraction (PDB ID: 8P4Z [169]). Both structures of CDK7 bound to LDC4297 show a nearly identical binding pattern with hydrogen bonding interactions with the backbone amide and carbonyl of CDK7 residue methionine 94 in the hinge region. Thus, the binding patterns of vCDK/pUL97 + MBV (Figure 11A) compared to CDK7 + LDC4297 (Figure 11B) reveal both similarities (i.e., spatial site of drug interaction with the ATP-binding pocket of the kinase domains) and differences (i.e., specific patterns of amino acid contact points). This information may support the further refined exploitation of these kinase targets for ongoing antiviral drug development.

## 6. Current Options of Pharmacological Enhancement of Kinase Inhibitors’ Antiviral Efficacy

### 6.1. ATP-Competitive, Substrate-Competitive, and Cyclin-Competitive vCDK Inhibitors

During the recent period of antiviral research, a number of different, and sometimes complementary, options of drug targeting have been strategically optimized. As far as kinase inhibitors are concerned in the classical way, the ATP-competitive mode of inhibition has been successful and could be applied on a broad basis, including the amazing success of kinase inhibitors in modern cancer therapies. Beyond that, however, more target-specific modes of action (MoA) of kinase inhibitors have been investigated. The substrate-competitive MoA is considered a very promising approach, in particular for applications in antiviral therapy. Here, it appears mostly beneficial to maintain the physiological role of a targeted, virus-supportive host kinase, by avoiding classical ATP-competitive inhibitors, instead trying to exclusively block the virus-specific functions of this given kinase. This requires, of course, knowledge about the interactive network of a kinase with viral proteins, i.e., phosphorylated viral substrate proteins first of all, and to develop pharmacological ways and novel compounds that specifically interfere with this type of virus–host interaction. A related inhibitory concept has been indicated by examples given in this review, namely cyclin-competitive inhibition of vCDKs. In the case of HCMV vCDK/pUL97, the importance of cyclin binding has been demonstrated by recent reports, so that this specific type of antiviral MoA appears accessible to drug development. First-model compounds have already been experimentally described in their antiviral efficacy, such as inhibitors of pUL97–cyclin H interaction that exert a pronounced antiviral activity [170]. Future studies will have to substantiate the value of these promising molecular options.

### 6.2. Mechanistical Enhancement of Investigational vCDK Inhibitors

In the attempts to develop antiherpesviral vCDK inhibitors, such as the lighthouse example of MBV for the treatment of HCMV disease, an extension of this strategy appears rewarding. At the moment, novel approaches are developed in order to transfer the success with the HCMV kinase inhibitors to other herpesviral kinase inhibitors, such as EBV BGLF4, VZV ORF66, or HHV-6 pU69. In this regard, it will be specifically interesting to see the potency of kinase inhibitors that may have an enhanced binding affinity to drug-resistance mutations of a given vCDK target. In the case of HCMV, this aspect is easily conceivable for GCV-resistance mutants that may show an enhanced sensitivity to MBV-like drug derivatives. Similar success has been made in the field of mutation-targeted anticancer therapies. Such a concept might particularly open the stage for covalently binding kinase inhibitors. Basically, covalent protein ligands have been avoided for a long period of time due to potential toxicity issues related to their limited target specificity. The advance of the field offered a deeper understanding of the binding mechanism, leading to effective design principles. As a result, it became easier to design target-specific covalent inhibitors, thereby minimizing the chance of unwanted side effects. Recently, covalently binding kinase inhibitors, referred to as warhead compounds, have attracted the specific interest of researchers and have already entered the various levels of clinical applications, including antiviral strategies. Even beyond this type of MoA, based on covalent drugs, there are even more new developments in the field, which may yield additional innovative options to broaden the entire scientific antiviral repertoire. When we are thinking about current examples of success in the pharmaceutical development of proteolysis targeting chimeras (PROTACs), i.e., compounds that contain target-degrading signal moieties, or dual-selective target binders, as well as drug-synergistic combinations as described above, the toolbox of ideas does not seem to be saturated at all. It is all about the question of which of these approaches may factually translate into clinical use, to possibly set novel standards of antiviral drug development and therapy practice.

## 7. Conclusions and Future Perspectives

This article focuses on a better understanding the cytomegalovirus vCDK/pUL97, as well as related HvPKs, in terms of deciphering the kinase-specific regulatory aspects of viral pathogenesis and and novel options of antiviral drug targeting. Molecular details of the formation of vCDK/pUL97–cyclin complexes, the phosphorylation of a variety of substrate proteins, and the importance of these regulatory activities for viral fitness, replication, and pathogenic spread have been discussed. In this way, a clear validation of the target vCDK/pUL97 for next-generation antiviral drugs has been provided, and the clinical approval of MBV strongly supports this strategic goal. As briefly presented in Section 6, the future perspectives may not remain restricted to classical types of kinase inhibitors, but may involve the current examples of covalent, degrading, dual-targeting or combined drug synergies, and even more. It will be exciting to see to which extent the immense experiences made in other therapeutical fields, such as anticancer, antoinflammation, and antimicrobial drug development, can provide a stimulatory impact on antiviral drug research, and vice versa. Probably, the diversity of choices in the advancement of kinase inhibitors is as wide as the target kinases’ functional complexity. Thus, future investigations will have to provide a confirmation of the kinase-based antiviral drug-targeting concept.

## Figures and Tables

**Table 1:** Target, EC_50_, CC_50_, and SI of a panel of CDK inhibitors.

Compound	Target	EC_50_ (µM) ^a^	CC_50_ (µM) ^b^	SI ^c^
abemaciclib	CDK4/6	6.43 ± 3.77	15.54 ± 5.78	2.42
AZD4573 ^d^	CDK9	-	0.35 ± 0.09	-
CDK2 Inh II	CDK2	5.17 ± 1.25	>100	19.34
CVT-313 ^d^	CDK2	-	5.57 ± 0.05	-
CYC065 ^d^	CDK2/9	-	<0.1	-
Dinaciclib ^d^	CDK1/2/5/9	-	<0.1	-
LDC4297	CDK7	0.0082 ± 0.0015	>1	121.95
Riviciclib ^d^	CDK1/4/9	-	1.64 ± 0.06	-
Samuraciclib ^d^	CDK7	-	0.50 ± 0.21	-
SEL120	CDK8	0.067 ± 0.027	5.90 ± 3.85	88.06
SY1365 ^d^	CDK7	-	<0.1	-
THAL-SNS032	CDK9	0.024 ± 0.0026	0.14 ± 0.06	5.83

^a^ EC_50_ against HCMV AD169-GFP was determined via GFP-based replication assay (identical conditions as described for Figure 6C); EC_50_ values represent the mean of at least two biological replicates each ± SD. ^b^ CC_50_ on mock-infected HFFs was determined via Neutral Red uptake assay; CC_50_ values represent the mean of at least two biological replicates each ± SD. ^c^ SI was calculated as CC_50_/EC_50_. ^d^ compound was excluded from further analysis.

**Table 2:** Antiviral drug-interpretation scheme according to Bliss independence checkerboard assay ^a^.

Compound Combination	95% Confidence Interval Synergy Volume (µM^2^%) ^b^			Interaction Type ^c^
	Positive	Negative	Sum	
MBV + GCV	0.0	−397.9	−397.9	antagonistic
abemaciclib + GCV	4.6	−76.5	−71.9	antagonistic
abemaciclib + LDC4297	389.8	−4.5	385.3	strongly synergistic
abemaciclib + MBV	0.1	−67.7	−67.6	antagonistic
TF27 + GCV	20.4	−19.7	0.7	additive
TF27 + LDC4297	10.0	−6.7	3.3	additive
TF27 + LMV	61.1	−22.3	38.8	additive
MBV + LDC4297	138.6	−1.6	137.0	strongly synergistic
CDK2 Inh II + MBV	394.3	−12.3	382	strongly synergistic
SEL120 + LDC4297	217.0	−0.0	217	strongly synergistic
THAL-SNS032 + SEL120	148.4	−2.0	146.4	strongly synergistic
CDK2 Inh II + LDC4297	0.0	−251.3	−251.3	antagonistic

^a^ For HCMV infection of HFFs, the reporter virus AG169-GFP was used, for a duration of 7 days, as measured by GFP-based replication assay described elsewhere [159]; ^b^ 95% confidence interval of at least three biological replicates per combination. ^c^ interpretation of synergy volume sum according to the following classification: below −100, strongly antagonistic; −100 to +50, additive; +50 to +100, moderately synergistic; above +100, strongly synergistic.

**Table 3:** Antiviral drug-interpretation scheme according to Loewe fixed-dose additivity assay.

Compound Combination	Ratio	Replicates ^a^	CI_wt_^b^	Interaction Type ^c^
MBV + GCV	1:1	3	2.88 ± 0.83	antagonistic
abemaciclib + LDC4297	100:1	2	1.03 ± 0.11	additive
abemaciclib + MBV	100:1	1	1.21	moderately antagonistic
abemaciclib + GCV	1:1	3	0.59 ± 0.08	synergistic
GCV + LDC4297	100:1	3	0.66 ± 0.19	synergistic
MBV + CDK2 Inh II	1:1	2	0.20 ± 0.13	strongly synergistic
MBV + LDC4297	50:1	2	0.48 ± 0.35	synergistic
MBV + LDC4297	100:1	2	0.48 ± 0.15	synergistic
SEL120 + CDV	1:1	1	0.31	synergistic
SEL120 + GCV	1:10	2	0.34 ± 0.07	synergistic
SEL120 + LDC4297	10:1	3	0.55 ± 0.06	synergistic
SEL120 + LMV	1:100	2	0.75 ± 0.05	moderately synergistic
SEL120 + THAL-SNS032	50:1	3	0.66 ± 0.10	synergistic
TF27 + GCV	1:100	2	1.37 ± 0.28	moderately antagonistic
THAL-SNS032 + LDC4297	1:1	3	0.62 ± 0.14	synergistic

^a^ Individual experimental replicates were performed in quadruplicate measurements (two wells each, two GFP samples each); ^b^ CI_wt_ was calculated as (0.1 × CI_50_ + 0.2 × CI_75_ + 0.3 × CI_90_ + 0.4 × CI_95_); ^c^ interpretation of CI_wt_ value according to the following classification: <0.1–0.3, strongly synergistic; 0.3–0.7, synergistic; 0.7–0.85, moderately synergistic; 0.85–0.9, slightly synergistic; 0.90–1.10, additive; 1.10–1.20, slightly antagonistic; 1.20–1.45, moderately antagonistic; 1.45–3.3, antagonistic; 3.3–>10, strongly antagonistic.

## Data Availability

The data presented in this study are published in the reports cited in the individual sections, and can be made available in further detail on request from the corresponding author.
